# Deviation from the balanced time perspective and depression and anxiety symptoms: the mediating roles of cognitive-behavioral emotion regulation in a cross-cultural model

**DOI:** 10.3389/fpsyt.2025.1452455

**Published:** 2025-02-07

**Authors:** Hamed Abdollahpour Ranjbar, Ayse Altan-Atalay, Mojtaba Habibi Asgarabad, Bulent Turan, Mehmet Eskin

**Affiliations:** ^1^ Department of Psychology, Koç University, Istanbul, Türkiye; ^2^ Department of Psychology, Faculty of Humanities and Social Sciences, Istinye University, Istanbul, Türkiye; ^3^ Department of Psychology, Kadir Has University, Istanbul, Türkiye; ^4^ Department of Psychology, Norwegian University of Science and Technology, Trondheim, Norway

**Keywords:** deviation from the balanced time perspective, cognitive and behavioral emotion regulation, depression, anxiety, moderated mediation, culture

## Abstract

**Background:**

Time perspective (TP) influences how individuals perceive and classify their past, present, and future, impacting their cognition, behavior, and psychological outcomes. Deviation from the balanced time perspective (DBTP) is associated with mental health problems (e.g., depression and anxiety). Emotion regulation (ER) encompasses cognitive and behavioral processes to regulate emotions, with maladaptive strategies like rumination and withdrawal linked to depression and anxiety. Despite extensive research on TP and ER, their joint impact, particularly in the context of depression and anxiety, and cultural differences remain underexplored.

**Method:**

Participants (*N* = 513 Iranian, *N* = 470 Turkish) completed self-report questionnaires on time perspective, cognitive and behavioral ER, anxiety, and depression symptoms. A moderated mediation model was assessed, incorporating the exogenous variable of DBTP, with ER strategies as mediators, and endogenous variables of depressive and anxiety symptoms. The model accounted for cultural variations in the paths as a moderator.

**Results:**

Significant associations were found between DBTP, ER strategies, depression, and anxiety symptoms. Mediation analyses revealed that both cognitive and behavioral ER strategies (except for adaptive behavioral ER strategies) significantly mediated the associations between DBTP and depression and anxiety. Additionally, multigroup analyses suggested that these mediating effects were consistent across Iranian and Turkish samples, with exceptions in adaptive cognitive ER strategies.

**Conclusion:**

The study highlights the crucial role of TPs and ER strategies in predicting anxiety and depression symptoms, with notable cultural nuances. Specifically, maladaptive strategies exacerbate symptoms, while adaptive strategies mitigate them primarily in Iranian contexts. Cultural subtleties are discussed in detail.

## Introduction

Cognitions and emotions are influenced by how we view and classify past, present, and future. Time perspective (TP) refers to an often non-conscious process through which experiences are categorized into time categories, aiding in organizing and making sense of these events (1). An important consideration is that TP often remains beyond conscious awareness. While we can recognize our current temporal focus, this realization occurs infrequently in practice. Zimbardo and Boyd (2) refer to this phenomenon as one of the key “time paradoxes.”

Five TP dimensions were established by Zimbardo and Boyd (1). Adverse and unpleasant view of the past is reflected in the Past-Negative (PN) dimension. The Past-Positive (PP) dimension reflects a fond and nostalgic perspective on past experiences (1). The Future (F) dimension primarily focuses on the future and objectives (1). The Present Hedonistic (PH) dimension is defined as a pleasure-seeking perspective characterized by a premium on the here and now. Present-Fatalistic (PF) embodies a fatalistic perspective on both present and future life circumstances. PF denotes a mindset that views the present as something to be borne with resignation since individuals are subject to the uncertainties of “fate” (2, p. 1278) and that the future is predestined and unaffected by individual efforts.

Zimbardo and Boyd (2) introduced the concept of Balanced Time Perspective (BTP) by presenting an optimal profile of time perspective, which includes low scores on PF and PN dimensions (10^th^ percentile of normative data), moderately high scores in PH and F dimensions (80^th^ percentile), and high scores in PP (90^th^ percentile), as measured by the Zimbardo Time Perspective Inventory (1). Research highlights the significance of cultivating a Balanced Time Perspective (BTP) (3, 4). The concept of an “ideal” optimal temporal perspective involves a dynamic interplay among attitudes toward the past, present, and future, adapting to situational demands, values, and individual needs (5). This construct is characterized by flexibility, prioritizing harmony over rigid norms, and has been shown to predict subjective well-being significantly across various studies (3, 6).

Researchers have developed three approaches to assess BTP. The initial indicator of balance was determined using the 33rd percentile cut-off point on the TP dimensions (7). To be considered balanced, individuals needed to score below the 33rd percentile in the PN and PF dimensions while scoring above this threshold in the other time perspectives. Later, Boniwell et al. (3) used a cluster-analysis method and identified four distinct profiles based on Zimbardo Time Perspective Inventory (ZTPI) scores: future-oriented, present-oriented, negative, and balanced. Stolarski et al. (8) introduced the Deviation from the Balanced Time Perspective (DBTP), which represents a *persistent* temporal bias that contrasts with the BTP (5). It refers to the extent to which an individual’s orientation toward past, present, and future perspectives deviates from an optimal or “balanced” time perspective (BTP). Zhang et al. (6) found DBTP to have the highest predictive validity for subjective well-being among the three methods. This indicator of temporal balance was developed using an analogy to Euclidean distance (see the methods section) and has been extensively studied in the context of well-being and mental health. DBTP has shown moderate to strong negative associations with various well-being aspects, accounting for up to 40% of the variance in well-being (9). Research shows DBTP is a substantial risk factor for a number of psychiatric conditions, such as major depression, generalized anxiety, obsessive-compulsive disorder (10, 11), and increased symptoms of depression, anxiety, stress, and negative mood (12–14).

Emotion regulation (ER) covers the intricate array of cognitive and behavioral processes employed in response to the different affective states (15). In navigating the intricacies of ER, it is important to discern between adaptive and maladaptive strategies. Hypothetically adaptive ER strategies comprise a spectrum of approaches to efficiently regulate emotions, promote psychological resilience, and foster positive outcomes (16). Conversely, putatively maladaptive ER strategies exacerbate psychological distress (17–20).

The deliberate cognitive processes employed to control emotionally charged information are termed cognitive ER (21). According to Garnefski et al. (21), there are marked differences between cognitive and behavioral processes involved in regulating emotions. Behavioral ER strategies have been found to be associated with symptoms of psychopathology, such as depression and anxiety (22, 23). Notably, withdrawal and ignoring demonstrate positive associations with both depressive and anxious symptoms (24).

Despite extensive studies on both topics, there is a gap in the literature on the associations between time perspective and emotion regulation (25). It has been suggested that one’s temporal orientations are intricately intertwined with their approach to regulating emotions (26). For instance, Bolotova and Hachaturova (26) showed a negative past orientation leads to emotional coping strategies, while a fatalistic present orientation results in retreat and avoidance of conflict resolution (27), which are maladaptive and involve fewer coping values (28, 29). Some research even suggests that time perspective can be a primary determinant of one’s current emotional state (e.g., 8, 30). On the other hand, the fluctuation in one’s current emotional state is a fundamental aspect and byproduct of emotion regulation (31). Therefore, the link between TPs and current emotional states might encompass emotion regulation.

Individuals frequently navigate through time dimensions—past, present, and future—in response to situational demands, emotional contexts, attitudes, and personal intentions (32). It is reasonable that individuals draw on different TPs when exploring solutions, especially in response to their internal emotional states. Therefore, when encountering challenges or stressors, people can reflect on past experiences to inform their judgments, consider present circumstances to assess immediate options, or project into the future to foresee potential outcomes. By optimal use of these TPs, individuals can better navigate their emotions and situations, ultimately leading to more effective problem-solving and emotion regulation. Given that emotion regulation is an integral aspect of self-regulation, it is possibly *intertwined* with time perspectives. This highlights the importance of further investigating how TP influences ER and their joint contribution to psychological outcomes.

### Culture, time perspective, and emotion regulation

There can be notable cross-cultural differences in individuals’ relationship with the concept of time (e.g., 33, 34). There have been few cross-cultural comparative investigations into TP using ZTPI (e.g., 3, 35, 36). The majority of these studies have focused on the cross-cultural consistency and universality of the ZTPI (see; 35), with findings indicating consistency (invariance) in ZTPI dimensions (i.e., PN, PP, PF, PH, and F). In their work with 24 cultures Sircova et al. (37) found significant patterns of similarity and differences in the prevalence of TP profiles across cultures. For instance, positive TP profiles were found to be the most prevailing patterns across cultures. Still, countries showed certain differences in specific TPs. For example, China had relatively high scores on the balanced and negative TP profiles, while Israel had very low scores on these dimensions (37, p. 183). In another study Sobol-Kwapińska et al. (38) compared the data from the United States, Poland, and Nigeria. Their findings indicated a similar pattern of perception of four dimensions except for PF, and Nigerian participants showed significant differences compared to the other two countries. The authors conclude that, in general, Nigerians, and likely Africans, have a tendency to perceive time as a holistic and less organized construct compared to Western cultures, warranting further investigation (38, p. 8). These findings underscore the possible variabilities between cultures, which can play a significant role in mental health outcomes.

Cultural variation also applies to emotion regulation, suggesting that cultural differences may potentially show up in the way people experience and deal with their emotions (39). A systematic review of studies shows that individualistic cultures promote emotional expression for emotion regulation, while collectivistic cultures support the usage of expressive suppression (40). Cultural factors can also impact the adaptivity of ER. For instance, expressive suppression has been associated with increased negative affect and depression within individualistic cultures such as those of the United States (41). Conversely, this association was not observed in collectivistic cultures such as Türkiye (42) and China (43).

Potthoff et al. (44) compared cognitive ER strategies in six European countries to examine if the association between specific ER strategies and psychopathology varies across cultures. Their findings indicated significant variations between northern and southern European countries. Northern Europeans exhibited a reduced use of strategies such as rumination, catastrophizing, and external blame. Additionally, “mean” differences in certain Cognitive Emotion Regulation Questionnaire (CERQ) strategies were identified between American and Chinese participants (45).

Considering the literature, further research is required to understand the scope of these variations between cultures. Exploring how individuals from diverse cultural backgrounds perceive and value the past, present, and future and how these perceptions influence their ER strategies, with downstream effects on depressive and anxiety symptoms, could provide valuable insights and support the ecological validity of theoretical perspectives.

### Current study

The present study investigates the relationships between DBTP and ER strategies, as well as symptoms of depression and anxiety, and examines how these associations vary between the cultures of Iran and Türkiye. According to Stolarski et al. (9), possessing a BTP could benefit individuals with temporal flexibility, enabling efficient mood/emotion regulation. Given the hypothesis, we suggest that the absence of BTP (i.e., DBTP) might potentially lead to a deterioration in ER and positive mood. This may result from either an upsurge in the use of maladaptive ER strategies or a decline in the use of adaptive ER strategies. To elucidate more, individuals with greater DBTP could find it difficult to keep their emotional equilibrium since they have a restricted ability to shift freely across time frames, which could make them more prone to psychological distress. This study aims to explore the potential mediating roles of ER strategies in the association between DBTP and symptoms of depression and anxiety, considering the possible cultural variations (i.e., the moderating role of culture). Thus, we hypothesized that I) DBTP would be positively associated with maladaptive cognitive-behavioral ER strategies, depression, and anxiety symptoms, II) DBTP would be negatively associated with adaptive cognitive-behavioral ER strategies, III) the association between DBTP and depression and anxiety symptoms would be mediated by adaptive/maladaptive cognitive-behavioral ER strategies, IV) These associations would differ between two cultures.

## Method

### Participants and procedure

Before collecting data, the Institutional Review Board (IRB) at Koç University granted ethical approval to ensure the protection of participants’ rights and welfare (IRB code: 2020. 427.IRB3.165). Participants were recruited through the Qualtrics platform, and prior to their involvement in the study, all were required to sign informed consent. They completed demographic details, including age, gender, academic field, academic level, self-reported psychiatric conditions, measures of variables of interest, and financial status. A total of 983 participants (513 Iranian and 470 Turkish) attended the study. The gender and age distribution of data included 301 males, 58.7%, with an age range of 18-63 (Mage = 30.35, SDage = 8.8) for the Iranian group and 119 males, 25.3%, with an age range of 18-64 (Mage = 25.65, SDage = 9.9) for the Turkish group.

### Measures

#### The Behavioral Emotion Regulation Questionnaire (BERQ)

The Behavioral Emotion Regulation Questionnaire (BERQ; 46) was developed to evaluate individuals’ behavioral responses to stressful life situations. This self-report questionnaire comprises 20 items and encompasses five distinct subscales: seeking distraction (e.g., *engaging in other activities*), withdrawal (e.g., *avoiding interactions with others*), actively approaching (e.g., *taking action to address the issue*), seeking social support (e.g., *seeking advice from someone*), and ignoring (e.g., *pretending as if nothing is happening*) (46). Each subscale comprises four items, and participants rate each item using a 5-point Likert scale ranging from 1 (*almost never*) to 5 (*almost always*). To obtain the total scale score for each subscale, the scores of the individual items are summed up, resulting in scores ranging from 4 to 20. Higher scores on the subscales indicate a higher tendency to utilize the corresponding emotion regulation strategy. We have used adapted versions of BERQ for both cultures (22, 23). For the current study, Cronbach’s α for the five subscales ranged from .81 to .85 (0.7 ≤ α< 0.8 indicates acceptable reliability).

#### The Cognitive Emotion Regulation Questionnaire (CERQ)

The Cognitive Emotion Regulation Questionnaire (CERQ; 47) was developed to evaluate individuals’ cognitive emotion regulation strategies when faced with challenging life circumstances. The CERQ is a 36-item self-report questionnaire that contains nine 4-item dimensions: self-blame (e.g., *I think about the mistakes I have made in this matter*), blaming others (e.g.*, I feel that others are responsible for what has happened*), acceptance (e.g.*, I think that I have to accept the situation*), refocusing on planning (e.g., *I think about how to change the situation*), positive refocusing (e.g.*, I think of pleasant things that have nothing to do with it*), rumination (e.g.*, I dwell upon the feelings the situation has evoked in me*), positive reappraisal (e.g.*, I think that the situation also has its positive sides*), putting into perspective (e.g.*, I think that other people go through much worse experiences*), and catastrophizing (e.g., *I keep thinking about how terrible it is what I have experienced*).

Participants rate their responses to items on a 5-point Likert scale, from 1 *“almost never”* to 5 *“almost always.”* We have used adapted versions of CERQ for both cultures (48, 49). In the current study, the Cronbach’s α ranged from.65 to.84.

#### Zimbardo Time Perspective Inventory (ZTPI)

Zimbardo Time Perspective Inventory (ZTPI; 1) has 56 items. ZTPI assesses individuals’ time orientation across five different temporal dimensions: Past Positive (e.g., “*It gives me pleasure to think about my past.”*), Past Negative (e.g., “*Painful past experiences keep being replayed in my mind”*), Present Hedonistic (e.g., “*I try to live my life as fully as possible, one day at a time”*), Present Fatalistic (e.g., “*Fate determines much in my life”*), and Future (e.g., “*It upsets me to be late for appointments”*). Participants respond to a 5-point Likert scale to rate the items, ranging from 1 for *“not at all”* to 5 for *“very much so.”* In the current study alpha values ranged between.74 and.82. In this study, we have used the 36-item version of ZTPI, which is deemed to be the “gold standard” for cross-cultural research (37, p. 183). We have used adapted versions of ZTPI for both cultures (50, 51).

#### Deviation From the Balanced Time Perspective (DBTP)

Deviation From the Balanced Time Perspective (DBTP; 8 **):** we employed the DBTP calculation suggested by (8). “This ZTPI-based indicator of temporal balance was developed *per analogiam* to Euclidean distance” (9, p 2):


$$DBTP=\sqrt{{\left(oPN-ePN\right)}^{2}+{\left(oPP-ePP\right)}^{2}+{\left(oPF-ePF\right)}^{2}+{\left(oPH-ePH\right)}^{2}+{\left(oF-eF\right)}^{2}}$$

DBTP is calculated by taking the square root of the sum of the squared deviations between an individual’s empirical (‘e’) mean scores on specific ZTPI scales and the ‘optimal’ (‘o’) points on each dimension. These optimal points are derived from Zimbardo and Boyd’s (2008) cross-cultural collective database and amounted to 4.6 for PP, 3.9 for PH, 4.0 for F, 1.95 for PN, and 1.5 for PF, reflecting 90th, 80th, 80th, 10th and 10th percentiles, respectively. For instance, if individual scores on five measurements of PN, PP, PF, PH, and F, are 2, 5, 4, 3, 4, respectively, their score of DBTP will be calculated like this:


$$DBTP=\sqrt{{\left(1.95-2\right)}^{2}+{\left(4.6-5\right)}^{2}+{\left(1.5-4\right)}^{2}+{\left(3.9-3\right)}^{2}+{\left(4.0-4\right)}^{2}}$$

$$\begin{array}{c} DBTP=\sqrt{0.0025+0.16+6.25+0.81+0}\\ DBTP=\sqrt{7.2225}\\ DBTP\approx 2.69 \end{array}$$

#### Patient Health Questionnaire-9 (PHQ-9)

Patient Health Questionnaire-9 (PHQ-9; 52) is used to assess individual differences in depressive symptoms. The PHQ-9 scale assesses each of the nine criteria for major depressive disorder outlined in the DSM-IV (Diagnostic and Statistical Manual of Mental Disorders, Fourth Edition). It asks respondents how often they have been affected by certain issues (e.g., “*feeling down, depressed, or hopeless*”) over the past two weeks, with answers given on a 4-point Likert scale ranging from 0 (*not at all*) to 3 (*nearly every day*). The total score can range from 0 to 27, with higher scores indicating greater severity of depression. Sarı et al. (53) conducted the Turkish validation study of the scale, revealing strong reliability with an alpha coefficient of.84. In the Iranian context, Dadfar et al. (54) also reported Cronbach’s alpha of.88. In the current study, Cronbach’s α was.88 (0.8 ≤ α< 0.9: indicates good reliability).

#### Generalized Anxiety Disorder-7 (GAD-7)

Generalized Anxiety Disorder-7 (GAD-7; 55) comprises seven items, with responses provided on a 4-point Likert-type scale (*1 = never, 4 = nearly every day*). This self-report measure evaluates anxiety severity over the previous two weeks in accordance with DSM-four criteria. The original scale demonstrated strong internal consistency (α = .92), and reliable test-retest reliability (r = .83) and exhibited convergent, criterion, and construct validities (55). Konkan et al. (2013) conducted validation of the Turkish version of the scale, revealing high internal consistency (α = .85) and providing evidence of construct validity (56). For the Iranian population Fattah et al. (57) reported the Cronbach’s alpha of.88. In the study at hand, the Cronbach’s alpha coefficient was calculated to be.91 [α ≥ 0.9: indicates excellent reliability (high internal consistency)].

### Statistical analysis procedure

The data were analyzed using SPSS 28.0.1 [IBM Corp, 2021 (58)] and Mplus 8.8 (59-2023), employing the following five steps. For preliminary analysis, all variables were checked for normality, linearity, and homoscedasticity based on conventional standards (60). Normality was assessed through skewness and kurtosis values. Visual inspection of Q-Q plots and histograms provided further support for the normality assumption. No significant outliers were detected. (See **Table 1** for more details and refer to Step 4 in this section for additional information). Given the adequacy of the sample size, no adjustments were deemed necessary (61).

**Table 1:** Bivariate correlations between deviation from the balanced time perspective, cognitive emotion regulation, behavioral emotion regulation depression, and anxiety.

	M	SD	IQR	1	2	3	4	5	6	Skewness	Kurtosis
1. DBTP	2.49 (2.39)	.77(.78)	1.03	1						.51 (.18)	.29 (-.13)
2. MBER	2.17 (2.62)	.70(.73)	1.04	.43^**^(.42^**^)	1					.72(.19)	.19 (-.16)
3. ABER	2.82 (3.46)	.66(.59)	1.01	-.23^**^(-.23^**^)	.04(-.04)	1				.37 (-.43)	.15 (1.29)
4. MCER	2.58 (2.99)	.61(.54)	.90	.43^**^(.43^**^)	.40^**^(.36^**^)	.11^*^(.16^**^)	1			.70 (-.29)	.45 (.80)
5. ACER	3.11 (3.35)	.68(.54)	.88	-.36^**^(-.29^**^)	.04 (.07)	.58^**^(.44^**^)	-.02(.04)	1		.04 (-.60)	-.58 (1.41)
6. Depression	17.50 (18.83)	6.70(6.17)	9	.58^**^(.51^**^)	.47^**^(.38^**^)	-.10^*^(-.02)	.53^**^(.47^**^)	-.26^**^(-.12^**^)	1	.79 (.51)	-.10 (-.30)
7. Anxiety	13.41 (14.77)	5.03(5.70)	7	.56^**^(.44^**^)	.40^**^(.28^**^)	-.09^*^(.02)	.53^**^(.48^**^)	-.29^**^(-.12^*^)	.80^**^(.76^**^)	.94 (.57)	.31 (-.55)

^**^ = Correlation is significant at.01 level (2-tailed). ^*^ = Correlation is significant at.05 level (2-tailed). IQR, Interquartile Range; DBTP, Deviation from the Balanced Time Perspective; MBER, Maladaptive Behavioral Emotion Regulation; ABER, Adaptive Behavioral Emotion Regulation; MCER, Maladaptive Cognitive Emotion Regulation; ACER, Adaptive Cognitive Emotion Regulation, Values out of the parentheses belong to Iran.

In the 2^nd^ step, the adequacy of the models was evaluated using various statistical tests and index values. These included the Comparative Fit Index (CFI), where coefficients greater than .95 indicate good fit (62); the Chi-square/degree of freedom ratio (CMIN/df), with values less than 5.0 suggesting good fit (63); the Tucker-Lewis index (TLI), with coefficients greater than .95 indicating good fit (64); and the Root Mean Square Error of Approximation (RMSEA), where values less than or equal to .08 suggest good fit (63, 65). Additionally, the Satorra-Bentler scaled chi-square test statistic was utilized to adjust the fit indices of all models and account for multivariate skewness in the data (66). The CFI and TLI are particularly useful for comparing models and assessing incremental fit, highlighting how well the proposed model improves upon a baseline model. The normalized Chi-square indicates a better balance between model complexity and fit, with smaller values reflecting a more parsimonious model. RMSEA evaluates the degree of discrepancy between the model and the population covariance matrix, indicating close fit while penalizing overly complex models. Finally, the Satorra-Bentler scaled chi-square adjusts for multivariate skewness and non-normality, ensuring robust and accurate estimation of fit indices in datasets with deviations from normality.

**Figure 1:**
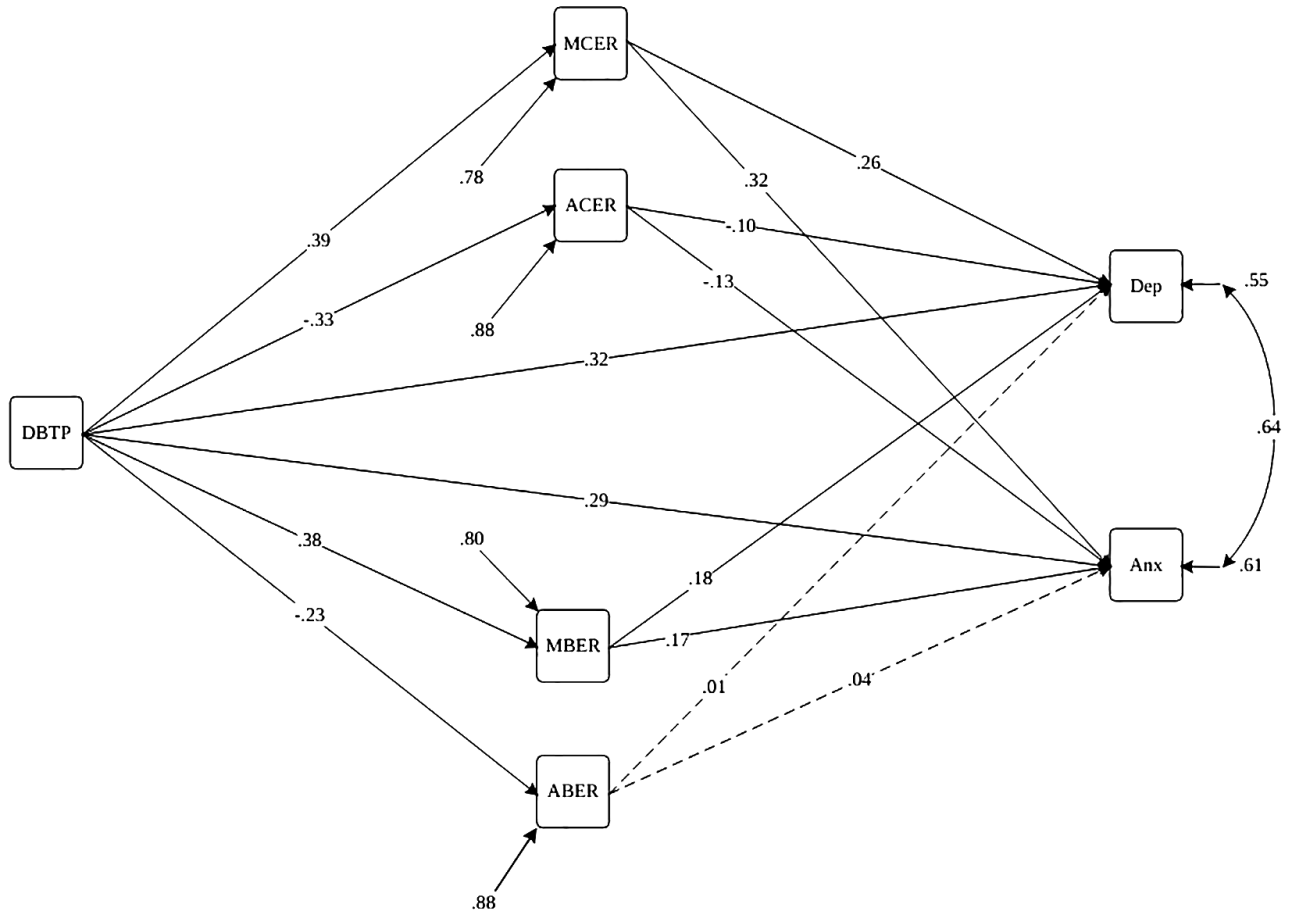
Path diagram of the structural model for Deviation from the balanced time perspective, cognitive-behavioral emotion regulation, and depression and anxiety symptoms. Note. Paths are statistically significant at p<.05 (dashed lines are insignificant paths). DBTP, Deviation from the Balanced Time Perspective; MBER, Maladaptive Behavioral Emotion Regulation; ABER, Adaptive Behavioral Emotion Regulation; MCER, Maladaptive Cognitive Emotion Regulation; ACER, Adaptive Cognitive Emotion Regulation; Anx, Anxiety Symptoms; Dep, Depression Symptoms.

In the 3^rd^ step, DBTP served as the exogenous variable in Model 1, with depression and anxiety symptoms as endogenous variables, maladaptive cognitive emotion regulation (MCER), maladaptive behavioral emotion regulation (MBER), adaptive cognitive emotion regulation (ACER), and adaptive behavioral emotion regulation (ABER) acting as mediators, as depicted in **Figure 1**.

In the 4^th^ step, the traditional indirect, direct, and total effects, commonly utilized in mediation research, along with their standard errors, were calculated using the MODEL INDIRECT command in Mplus 8.8 (67). A significant indirect effect suggests mediation. It’s important to note that the mediator model was evaluated using maximum likelihood estimation (MLR) with robust standard errors (68) in both models 1 and 2. Indirect effects were examined using bias-corrected bootstrap methodology with 100,000 replications and 95% confidence intervals (CIs) to assess significance (62). The use of 100,000 bootstrap replications was chosen to ensure precise and reliable estimates of indirect effects. Research shows that higher replication counts reduce Monte Carlo (MC) error and variability in confidence intervals, particularly in complex models or small samples (69–71).

**Figure 2:**
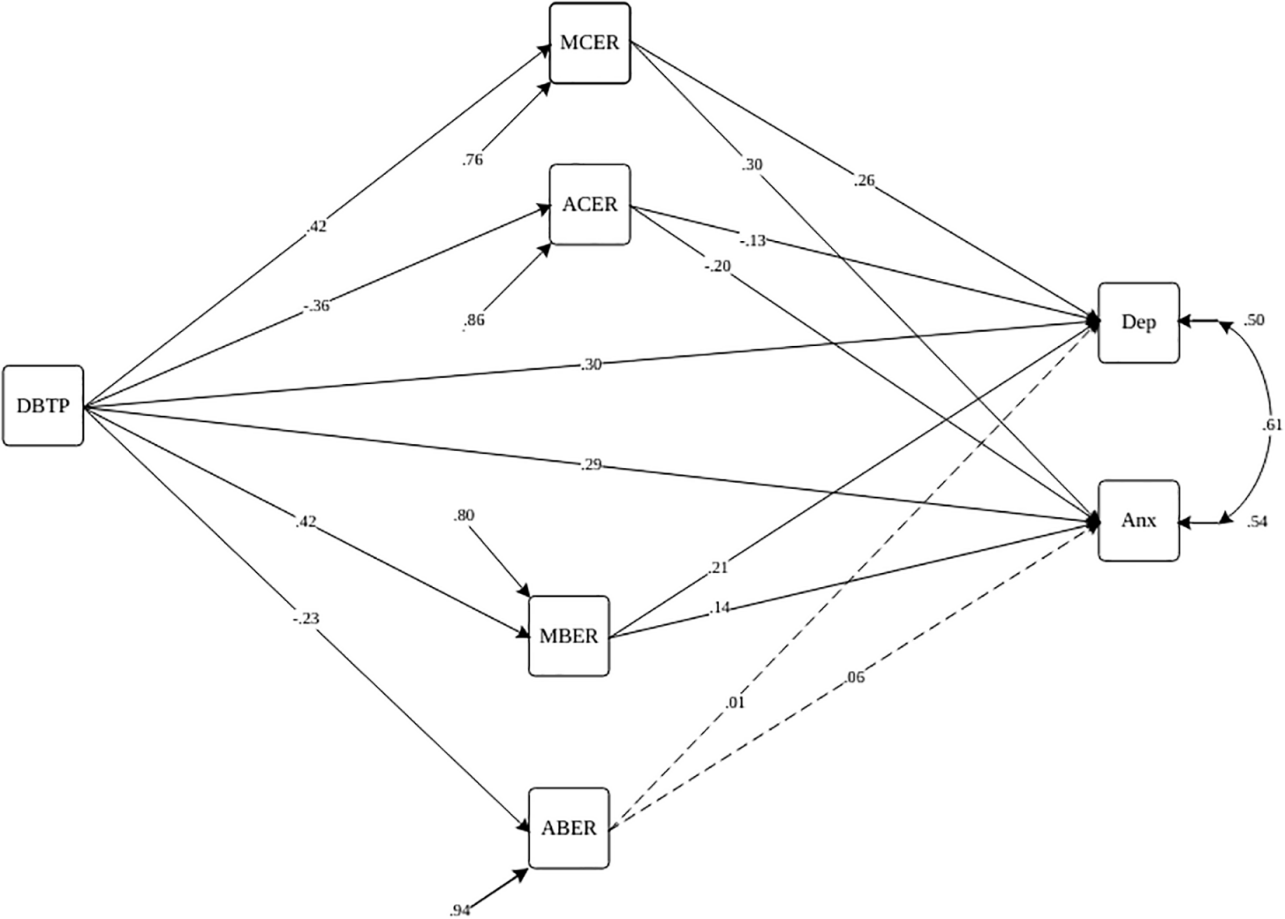
Path diagram of the structural model for Deviation from the balanced time perspective, cognitive-behavioral emotion regulation, and depression and anxiety symptoms with the moderating role of culture (Iran) Note. Paths are statistically significant at p<.05 (dashed lines are insignificant paths). DBTP, Deviation from the Balanced Time Perspective; MBER, Maladaptive Behavioral Emotion Regulation; ABER, Adaptive Behavioral Emotion Regulation; MCER, Maladaptive Cognitive Emotion Regulation; ACER, Adaptive Cognitive Emotion Regulation; Anx, Anxiety Symptoms; Dep, Depression Symptoms.

**Figure 3:**
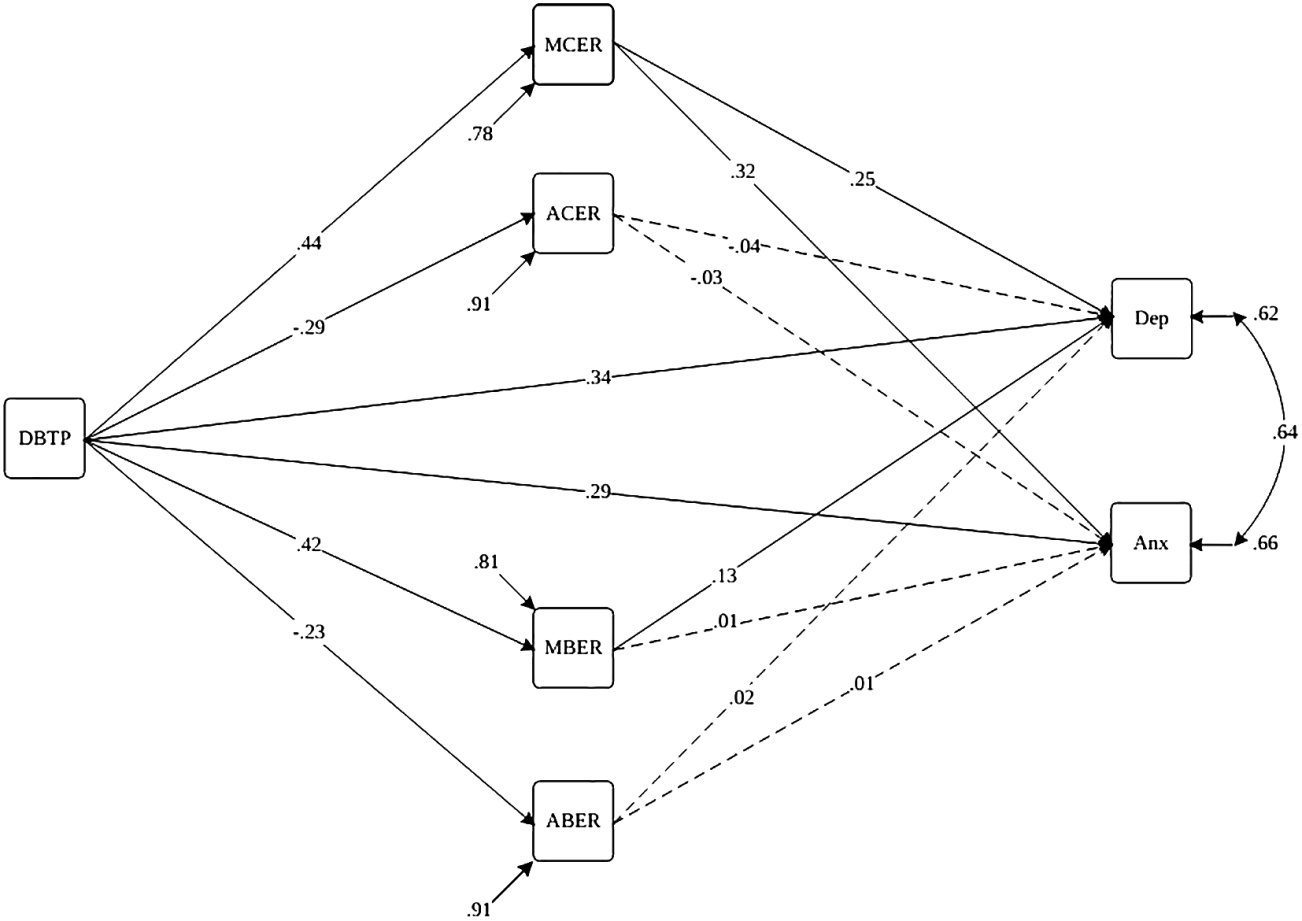
Path diagram of the structural model for Deviation from the balanced time perspective, cognitive-behavioral emotion regulation, and depression and anxiety behavior with the moderating role of culture (Türkiye) Note. Paths are statistically significant at p<.05 (dashed lines are insignificant paths). DBTP, Deviation from the Balanced Time Perspective; MBER, Maladaptive Behavioral Emotion Regulation; ABER, Adaptive Behavioral Emotion Regulation; MCER, Maladaptive Cognitive Emotion Regulation; ACER, Adaptive Cognitive Emotion Regulation; Anx, Anxiety Symptoms; Dep, Depression Symptoms.

In the 5^th^ step, we simultaneously assessed the moderation model alongside the mediation model (Model 2, **Figures 2**, **3**) to investigate cultural distinctions between Iran and Türkiye. Our examination centered on a moderated mediation model, exploring the relationship between DBTP and depression and anxiety, with cognitive-behavioral emotion regulation strategies acting as mediators for both countries. To investigate the moderating role of culture in our mediation model, we conducted a moderated multi-group mediation analysis. In this analysis, culture is treated as a dichotomous moderator, coded as 0 and 1 to represent two distinct cultural groups. The cultural groups were dummy coded as follows: Culture 0 represented Group A (Iranian culture). Culture 2 represented Group B (Turkish culture). Rather than examining the classic interaction effects in a single regression model, we ran separate regression models for each cultural group. This approach allows comparing the regression coefficients across the two groups directly. Separate mediation models were specified for each cultural group. Key paths in the mediation model were estimated for each group independently. Fit indices for each group-specific model were evaluated. If a model demonstrated a poor fit for one or both groups, it suggested potential moderation by culture. The Satorra-Bentler scaled chi-square difference test was employed to compare the fit of the constrained model (assuming equal path coefficients across groups) against the unconstrained model (allowing path coefficients to vary between groups). A significant chi-square difference test indicates that culture moderates the relationship, suggesting that path coefficients differ significantly across cultural groups. The sample exhibited a gender imbalance, particularly among Turkish participants, with a notably higher participation of women. Such trends are observable in various large-scale studies examining symptoms of depression and anxiety (72, 73). Thus, age, gender, and self-reported psychiatric diagnoses were incorporated as covariates to account for their potential influence on outcome measures.

## Results

**Table 1** presents descriptive statistics and pairwise correlations between DBTP, adaptive/maladaptive cognitive emotion regulation (A/M CER) indicators, adaptive/maladaptive behavioral emotion regulation (A/M BER) indicators, depression, and anxiety. The table illustrates that all correlations among exogenous, endogenous, and mediator variables in the overall sample are statistically significant (p<.05). These findings indicate strong conceptual and statistical support for the proposed causal model in mediation analysis. Therefore, we proceeded with examining a latent variable mediation model utilizing observed variables.

### Mediation analyses

The goodness-of-fit for the model 1 is presented in **Table 2**. A theory-driven specified model (M_1_ in **Table 2**, **Figure 1**; S-B χ^2^ = 23.57; p = .0001; CFI = .99; TLI = .93; and RMSEA = .07 ([CI] 95% = .045 to.099) meet the established fitting criteria.

**Table 2:** Modification indices for the mediated model of DBTP and cognitive-behavioral emotion regulation on depression and anxiety.

Model	S-B χ^2^(p value)	df	χ^2^/df	SCFMLR	CFI	TLI	RMSEA	SRMR
M1	23.575(.0001)	4	5.89	1.0063	.99	.93	.071(.045 -.099)	.019
M2	20.509(.0072)	8	2.56	1.0232	.95	.95	.049 (.042 -.056)	.064

M_1_ = structural equation modeling of pathways from DBTP to Depression and Anxiety: mediating cognitive/behavioral emotion regulation; M_2_ incorporates the moderation effect of country (Iran and Türkiye) on the model represented by M1. S-B *χ^2^* = the Satorra-Bentler scaled chi-square, df = degrees of freedom, *χ^2^*/df = normal chi-square, TLI, Tucker–Lewis index; SCFMLR, scaling correction factor for MLR; CFI, comparative fit index; SRMR,= standardized root mean square residual; RMSEA, root mean square error of approximation. In both models, we accounted for the potential influence of age, gender, and psychiatric diagnosis by including them as covariates. ^∗^ *p*<.05, ^∗∗^ *p*<.01.

The findings presented in **Table 3** highlight a significant direct association between DBTP (β = .32, p<.001), MCER (β = .26, p<.001), MBER (β = .18, p<.01), ACER (β = -.10, p<.01), age (β = -.14, p<.001), and psychiatric diagnosis (β = -.06, p<.05) with depression. Moreover, the table indicates a significant direct association between DBTP (β = .29, p<.001), MCER (β = .32, p<.001), MBER (β = .17, p<.05), ACER (β = -.13, p<.01), and age (β = -.14, p<.001) with anxiety. Additionally, DBTP demonstrates a significant direct effect on MCER (β = .39, p<.001), MBER (β = .38, p<.01), ACER (β = -.33, p<.01), and ABER (β = -.23, p<.001). Furthermore, anxiety and depression symptoms are observed to have a significant cross-sectional association as outcome factors of the model (β = .64, p<.001).

**Table 3:** Standardized direct effects of DBTP and cognitive-behavioral emotion regulation on depression and anxiety using the bootstrap method.

Paths	Direct effect	*p*	95% CI
MCER→ Depression	**.26**	.001	[.24.33]
MBER → Depression	**.18**	.001	[.12.24]
ACER → Depression	**-.10**	.002	[-.17 -.02]
ABER → Depression	.01	.889	[-.06.07]
DBTP → Depression	**.32**	.001	[.24.38]
Age → Depression	**-.14**	.001	[-.19 -.09]
Gender → Depression	.03	.205	[-.02.08]
Psychiatric diagnosis → Depression	**-.06**	.013	[-.12 -.01]
MCER→ Anxiety	**.32**	.001	[.26.39]
MBER → Anxiety	**.17**	.027	[.01.14]
ACER → Anxiety	**-.13**	.001	[-.19 -.07]
ABERQ → Anxiety	.04	.225	[-.02.11]
DBTP → Anxiety	**.29**	.001	[.21.35]
Age → Anxiety	**-.14**	.001	[-.19 -.09]
Gender → Anxiety	-.01	.837	[.06.04]
Psychiatric diagnosis → Anxiety	-.04	.116	[-.10.01]
DBTP → MCER	**.39**	.001	[.31.44]
DBTP → MBER	**.38**	.001	[.32.43]
DBTP → ACER	**-.33**	.001	[-.39 -.27]
DBTP → ABER	**-.23**	.001	[-.30 -.16]
^†^MCER with MBER	**.30**	.001	[.23.37]
^†^MCER with ACER	**.23**	.001	[.16.31]
^†^MCER with ABER	**.35**	.001	[.28.40]
^†^MBER with ACER	**.28**	.001	[.22.34]
^†^MBER with ABER	**.20**	.001	[.13.27]
^†^ACER with ABER	**.52**	.001	[.47.57]
Depression with Anxiety^††^	**.64**	.001	[.59.68]

CI, confidence intervals; †, standardized covariance coefficients between mediator factors; ††, standardized covariance coefficients between endogenous factors. Bold font: indicates significant path. We included age, gender, and self-reported psychiatric diagnoses as covariates to account for their potential impact on the outcomes. DBTP, Deviation from the Balanced Time Perspective; MBER, Maladaptive Behavioral Emotion Regulation; ABER, Adaptive Behavioral Emotion Regulation; MCER, Maladaptive Cognitive Emotion Regulation; ACER, Adaptive Cognitive Emotion Regulation

The results from **Figure 1**, **Tables 4**, **5** indicate that MCER (β for indirect effect = .10, p<.001), ACER (β for indirect effect = .03, p<.01), and MBER (β for indirect effect = .07, p<.001) serve as mediators in the relationship between DBTP and depression. Additionally, MCER (β = .12, p<.001), ACER (β = .04, p<.01), and MBER (β = .03, p<.05) also acted as mediators in the association between DBTP and anxiety.

**Table 4:** Indirect standardized effects of DBTP and cognitive-behavioral emotion regulation on depression and anxiety using the bootstrap method.

	Estimation	SE	T-value	P	95% CI
Effects from DBTP to Depression					
Total Effect	**.52**	.028	18.64	.001	[.46,.57]
Total Indirect	**.20**	.02	9.95	.001	[.16,.25]
DBTP → MCER → Depression					
Indirect Effect	**.10**	.01	7.52	.001	[.08,.13]
DBTP → ACER→ Depression					
Indirect Effect	**.03**	.01	3.01	.003	[.01,.16]
DBTP → MBER→ Depression					
Indirect Effect	**.07**	.01	5.71	.001	[.04,.09]
DBTP → ABER → Depression					
Indirect Effect	-.01	.01	-.14	.88	[-.02,.01]
Effects from DBTP to Anxiety					
Total Effect	**.47**	.03	15.33	.001	[.41,.53]
Total Indirect	**.19**	.02	9.63	.001	[.14,.23]
DBTP → MCER → Anxiety					
Indirect Effect	**.12**	.02	8.01	.001	[.09,.16]
DBTP → ACER→ Anxiety					
Indirect Effect	**.04**	.01	3.68	.001	[.02,.06]
DBTP → MBER→ Anxiety					
Indirect Effect	**.03**	.01	2.20	.028	[-.03,.01]
DBTP → ABER → Anxiety					
Indirect Effect	-.01	.01	-1.22	.22	[-.03,.00]

Bold font: indicates significant path. DBTP, Deviation from the Balanced Time Perspective; MBER, Maladaptive Behavioral Emotion Regulation; ABER, Adaptive Behavioral Emotion Regulation; MCER, Maladaptive Cognitive Emotion Regulation; ACER, Adaptive Cognitive Emotion Regulation

**Table 5:** Standardized direct effects of DBTP and cognitive-behavioral emotion regulation on depression and anxiety using the bootstrap method ACROSS Iran and Türkiye.

Paths	Direct effect	*p*	95% CI
MCER→ Depression	**.26(25)**	.001(.001)	.17, .35(.15, .34)
MBER → Depression	**.21(.13)**	.001(.004)	.13, .29(.04, .23)
ACER → Depression	**-.13**(-.04)	.005(.40)	-.22, -.02(-.13, .05)
ABER → Depression	.01(.02)	.93(.65)	-.09, .09(-.07, .10)
DBTP → Depression	**.30(.34)**	.001(.001)	22, .40(.23, .44)
Age → Depression	**-.13(-.15)**	.001(.001)	-.19, -.07(-.24, -.10)
Gender → Depression	.05(-.04)	.11(.40)	-.02, .11(-.12, .04)
Psychiatric diagnosis → Depression	**-.09**(-.02)	.008(.59)	-.16, -.03(-.10, .06)
MCER→ Anxiety	**.30(.32)**	.001(.001)	.21, .39(.24, .42)
MBER → Anxiety	**.14**(.01)	.002(.88)	.05, .22(-.09, .10)
ACER → Anxiety	**-.20**(-.03)	.001(.47)	-.29, -.11(-.12, .06)
ABER → Anxiety	.06(.01)	.19(.82)	-.03, .14(-.09, .10)
DBTP → Anxiety	**.29(.29)**	.001(.001)	.19, .37(.20, .39)
Age → Anxiety	**-.07(-.20)**	.03(.001)	-.13, -.01(-.27, -.16)
Gender → Anxiety	.04(-.08)	.23(.05)	-.03, .10(-.16, .01)
Psycological Problem → Anxiety	**-.09**(.02)	.01(.63)	-.17, -.03(-.06, .11)
DBTP → MCER	**.42(.44)**	.001(.001)	.32, .50(.30, .53)
DBTP → MBER	**.42(.42)**	.001(.001)	.33, .49(.32, .50)
DBTP → ACER	**-.36(-.29)**	.001(.001)	-.45, -.27(-.39, -.19)
DBTP → ABER	**-.23(-.23)**	.001(.001)	-.34, -.13(-.33, -.13)
^†^MCER with MBER	**.24(.21)**	.001(.001)	.14, .33(.09, .35)
^†^MCER with ACER	**.20(.20)**	.001(.009)	.10, .30(.05, .35)
^†^MCER with ABERQ	**.22(.28)**	.001(.001)	.13, .31(.17, .41)
^†^MBER with ACER	**.25(.25)**	.001(.001)	.16, .33(.14, .35)
^†^MBER with ABERQ	**.15**(.04)	.001(.57)	.06, .24(-.08, .16)
^†^ACER with ABERQ	**.56(.42)**	.001(.001)	.49, .62(.33, .52)
Depression with Anxiety^†^	**.61(.64)**	.001(.001)	17, .35(.59, .69)

CI = confidence intervals. † = standardized covariance coefficients between mediator factors. †† = standardized covariance coefficients between endogenous factors. Bold font: indicates significant path. We included age, sex, and self-reported Psychiatric diagnoses as covariates to account for their potential impact on the outcomes. DBTP, Deviation from the Balanced Time Perspective; MBER, Maladaptive Behavioral Emotion Regulation; ABER, Adaptive Behavioral Emotion Regulation; MCER, Maladaptive Cognitive Emotion Regulation; ACER, Adaptive Cognitive Emotion Regulation. Values out of the parentheses belong to Iran.

### Moderation effect of country

We performed multigroup mediation analyses to investigate the moderating effect of country (Iran and Türkiye) on the mediation model outlined in the preceding steps and to evaluate the similarities and discrepancies in coefficients across the two countries. The goodness-of-fit indices for the moderator model are presented in **Table 2**, with the S-B *χ* ^2^ = 20.51, p = .0072, CFI = .95, TLI = .95, and RMSEA = .05 (95% CI = .042 to.056), all meeting specified fitting criteria. The chi-square difference test indicates that M_2_ does provide a better fit to the data than M_1_ (*Δχ* ^2^ = 3.06, *Δ*df =−4, p = .54).

In the Iranian sample (**Figure 2**, **Table 6**), MCER (β = .11, p<.001), ACER (β = .05, p<.01), and MBER (β = .09, p<.001) mediated the link between DBTP and depression. Similarly, MCER (β = .13, p<.001), ACER (β = .13, p<.01), and MBER (β = .06, p<.05) mediated the association between DBTP and anxiety.

**Table 6:** Indirect standardized effects of DBTP and cognitive-behavioral emotion regulation on depression and anxiety using the bootstrap method across Türkiye and Iran.

	Estimation	SE	T-value	P	95% CI
Effects from DBTP to Depression					
Total Effect	**.55(.51)**	.04(.05)	15.58(11.34)	.001(.001)	.48, .61 (.43, .59)
Total Indirect	**.24(.17)**	.03(.03)	8.28(5.52)	.001(.001)	.18, .30 (.11, .23)
DBTP → MCER → Depression					
Indirect Effect	**.11(.11)**	.02(.02)	5.78(5.21)	.001(.001)	.07, .15 (.07, .16 )
DBTP → ACER→ Depression					
Indirect Effect	**.05**(.01)	.02(.01)	2.77(.85)	.006(.40)	.01, .08 (-.02, .04)
DBTP → MBER→ Depression					
Indirect Effect	**.09(.05)**	.02(.02)	4.52(2.85)	.001(.004)	.05, .13 (.02, .10)
DBTP → ABER→ Depression					
Indirect Effect	-.01(-.01)	.01(.01)	-.09(-.44)	.93(.66)	-.02, .02 (-.03, .02)
Effects from DBTP to Anxiety					
Total Effect	**.53(.44)**	.04(.05)	14.28(9.26)	.001(.001)	.40, .59 (.35, .53)
Total Indirect	**.24(.15)**	.03(.04)	8.35(4.37)	.001(.001)	.19, .30(.09, .22)
DBTP → MCER → Anxiety					
Indirect Effect	**.13(.14)**	.02(.02)	6.04(5.97)	.001(.001)	.09 .17 (.10, .20)
DBTP → ACER→ Anxiety					
Indirect Effect	**.07**(.01)	.02(.01)	3.88(.72)	.001(.48)	.04, .12 (-.02, .04)
DBTP → MBER→ Anxiety					
Indirect Effect	**.06**(.01)	.02(.02)	3.02(.16)	.003(.17)	.02, .10 (-.04, .04)
DBTP → ABER→ Anxiety					
Indirect Effect	-.01(-.01)	.01(.01)	-1.28(-.22)	.20(.82)	-.04, .01 (-.03, .02)

Bold font indicates significant path.

In the Turkish sample (**Figure 3**, **Table 6**), MCER (β = .11, p<.001) and MBER (β = .05, p<.001) mediated the relationship between DBTP and depression, while only MCER (β = .14, p<.001) mediated the association between DBTP and anxiety. Notably, MBER did not serve as a mediator for anxiety symptoms in the Turkish sample. These results underscore the cultural differences in the mediation pathways, with MBER playing a more pronounced role in the Iranian context.

## Discussion

The way individuals attend to different time frames (e.g., DBTP) can influence their emotional experiences (e.g., depression; 30). In this study, we found that DBTP is associated with maladaptive cognitive and behavioral emotion regulation (ER) strategies, which mediate the relationship between DBTP and symptoms of depression and anxiety. Maladaptive cognitive ER mediated this relationship in both Turkish and Iranian contexts, while maladaptive behavioral ER mediated only depressive symptoms in both cultures. Adaptive cognitive ER mediated depression and anxiety only in the Iranian group, and adaptive behavioral ER showed no mediation effects in either culture. These findings highlight cultural differences in emotion regulation and their influence on mental health outcomes.

Our findings suggest a positive association between DBTP, maladaptive cognitive-behavioral ER strategies, and symptoms of anxiety and depression. Also, our results indicate a negative association between DBTP and adaptive cognitive-behavioral ER strategies and a positive association with depression and anxiety symptoms. These associations persisted consistently across both cultural contexts. The findings align closely with the extensive body of research demonstrating the significant relationship between DBTP and symptoms of depression and anxiety across diverse cultural settings (10, 74; Ranjbar et al., 2023).

Furthermore, we found that maladaptive cognitive emotion regulation (MCER) strategies can mediate the association between DBTP and both depressive and anxious symptomology, and this mediation effect is observed in both Turkish and Iranian cultures. These results suggest that individuals with higher levels of DBTP (e.g., high PN, high PF) may potentially experience symptoms of depression and anxiety in a more intense manner through frequent use of MCER strategies like rumination, catastrophizing, or blaming attitudes. This finding is in line with parallel conceptualizations from the literature. For instance, Bolotova and Hachaturova (26) demonstrated a link between TP profiles and cognitive coping strategies. Similarly, Bürhan Çavuşoğlu et al. (25) observed a positive association between maladaptive time perspectives like PN and PF and indicators of difficulties in emotion regulation. In a similar vein, Glazer et al. (75) found that the positive relationship between PN and rumination does not contribute to optimal psychological functioning in individuals.

Our findings indicate that maladaptive behavioral emotion regulation (MBER) strategies mediate the relationship between DBTP and both depressive and anxious symptoms. However, this mediation effect was found only for depressive symptoms in both Turkish and Iranian cultures. Specifically, MBER strategies were found to mediate the association between DBTP and anxiety symptoms in the Iranian but not the Turkish sample. These findings may indicate that individuals with higher levels of DBTP can potentially experience symptoms of depression and anxiety due to resorting to MBER strategies like withdrawal and ignoring. These findings are consistent with previous research on behavioral emotion regulation, particularly within the contexts of Turkish (23) and Iranian cultures (22), which has explored the associations between behavioral emotion regulation strategies and the manifestation of depressive and anxious symptoms.

Our findings suggest that there is a significant mediator role of adaptive cognitive emotion regulation (ACER) strategies in the relationship between DBTP and symptoms of anxiety and depression. However, when the moderating impact of culture was considered, this mediation effect persisted to remain statistically significant only in the Iranian group. This suggests that Iranian individuals who have a more balanced time perspective tend to employ ACER strategies more prominently or more effectively. These findings parallel prior studies on cognitive ER and psychological distress. Notably, Tuna and Bozo (49) examination of the Turkish cohort elucidated negligible and nearly non-existent associations between ACER strategies and various indices of psychological distress, encompassing depressive, anxious, and somatic symptoms (p. 568). In stark contrast, the investigation by Hasani et al. (76) within the Iranian population demonstrated strong negative associations between nearly all ACER strategies and indicators of psychological distress, including depression, anxiety, and stress (p. 6).

These results point to significant differences between the two cultural settings, which require further in-depth investigation. Some elaborative information can be provided by comparing these results with those of other contextual, cross-cultural research. More specifically, the correlations observed in Türkiye have similarities to those observed in Western nations. For example, Potthoff et al. (44) demonstrated modest correlations between psychological distress indicators and ACER strategies in their study of six European nations. Nonetheless, Megreya et al. (77) found strong negative correlations between negative affect (measured by PANAS) and ACER strategies in their study, including four Arab-speaking cultures (p. 886), which is consistent with the associations found in Iranian culture.

Psychological and cultural theories may also help explain the effective use of ACER strategies among Iranian individuals. The greater associations shown in the Iranian group may also be attributable to cultural norms that value emotional control and resilience, which may help individuals become more skilled in using ACER strategies to regulate their negative thoughts and feelings. In other words, the Iranian culture’s focus on emotional restraint and control (78) may promote the development and use of ACER strategies, increasing their effectiveness in reducing anxiety and depression while also more effectively satisfying psychological demands for relatedness, autonomy, and competence (see self-determination theory; 79). The internalization of these ACER strategies could be aided by the emotional socialization process in Iranian culture (80), which places a strong emphasis on the control and appropriate expression of emotions. Individuals acquire the ability to regulate their emotions from an early age, which is attributable to this cultural focus on emotional control, which promotes more efficient emotional regulation. This socialization process, in turn, may improve the utilization of ACER strategies to reduce psychological distress and promote mental health in accordance with cultural norms and values. Furthermore, Iranian individuals may be subject to more stringent social norms and perceived control, which may strengthen the efficacy of these strategies. Hofstede’s cultural dimensions theory also indicates that collectivist cultures, such as Iran (at least more collectivistic than Türkiye), emphasize social harmony and community support, which likely enhances the use of ACER strategies, leading to reduced anxiety and depression.

Our findings also indicate that adaptive behavioral emotion regulation (ABER) strategies do not mediate the relationship between DBTP and symptoms of anxiety and depression in either cultural context. This suggests that in both Turkish and Iranian cultures, ABER strategies—seeking distraction, seeking social support, and actively approaching—do not alleviate the impact of DBTP on anxiety and depressive symptoms. These observations could be due to some reasons. DBTP might involve profound cognitive and emotional patterns that are not easily addressed by surface-level coping mechanisms. ABER strategies, while helpful in general stress and emotion regulation, might not be specifically equipped to tackle the complex, long-standing issues stemming from DBTP. Strategies like seeking distraction or social support may provide temporary relief but might not address the underlying cognitive distortions associated with an unbalanced time perspective. Relevant interventional study findings also show the scenario-based adaptability of these strategies. For instance, Gebhart et al. (81), in their randomized controlled trial (RCT) investigating the impact of distraction-focused interventions on examination stress, demonstrated that the effectiveness of these strategies varied depending on the specific situational context.

Lastly, adaptive emotion regulation strategies go beyond what the BERQ and CERQ assess. The BERQ and the CERQ are useful instruments, but they only cover a portion of the wide range of strategies people employ to regulate their emotions when confronting stressful situations. Other strategies—mindfulness exercises, acceptance (82), and problem-solving techniques (83), self-compassion (84)—all have a significant impact on how individuals manage stress and emotional difficulties. Comprehending the entire range of these strategies can provide a more profound understanding of emotional resilience, underscoring the need to adopt a holistic approach to researching and assisting with emotion regulation.

### Limitations and future directions

A number of limitations must be acknowledged. The cross-sectional and observational design limits our ability to deduce causation. Thus, longitudinal and/or experimental research is required to gain a deeper understanding of these processes. Furthermore, biases like social desirability or recollection oversights may be introduced into self-reported assessments, which might affect the veracity of reported ER strategies and psychological symptoms. The use of experience sampling methods in subsequent research may be useful. The results may be impacted by cultural differences in how emotions are expressed and understood, underscoring the need for more sophisticated, culturally relevant measuring techniques and instruments.

Furthermore, the study’s emphasis on Turkish and Iranian cultures restricts the findings’ applicability to other cultural contexts, highlighting the necessity for cross-cultural research including a wider variety of cultures. We have assessed the history of psychiatric diagnosis and symptoms of depression and anxiety using self-report measures. However, this assessment is susceptible to the risk of bias, and future studies should focus on more objective assessments like structured clinical interviews. The present study has concentrated solely on symptoms of depression and anxiety. Subsequent research endeavors can broaden our paradigms and framework to encompass the domain of personality traits and explore various personality dimensions.

The findings have clinical relevance, suggesting that targeted interventions addressing time  perspective distortions and maladaptive ER patterns could potentially lead to improved outcomes. Further studies should investigate the cross-cultural generalizability of the findings by examining various populations, thereby ensuring that interventions are sensitive to and can be adapted across a range of sociocultural settings. Additionally, future studies might extend the proposed model further to include potential moderators, such as trauma history, social support systems, and personality factors, which would elucidate the heterogeneity in the relationships from DBTP to mental health results. Such variability in individual differences has prompted an emphasis on tailored mental health interventions that take cultural and psychosocial differences into consideration when diagnosing and treating depression and anxiety using psychosocial approaches.

## Conclusion

Our research sheds light on the complex links between DBTP, cognitive-behavioral ER strategies, and anxiety and depression symptoms in Turkish and Iranian cultural contexts. Strong associations were found between DBTP and maladaptive cognitive-behavioral ER strategies, as well as anxiety and depressive symptoms, highlighting the psychological processes’ broad applicability. Additionally, our results demonstrate the mediator function of maladaptive behavioral and cognitive ER strategies in the association between DBTP and depressive/anxious symptoms and with adaptive cognitive ER strategies acting as a mediating factor mainly within the Iranian cultural setting.

Moreover, our findings elucidate the nuanced nature of adaptive and maladaptive ER strategies, suggesting that while maladaptive strategies may contribute to the exacerbation of symptoms of anxiety and depression, adaptive strategies may not consistently alleviate these across both cultural contexts. This underscores the importance of considering cultural influences on coping mechanisms and mental health outcomes.

Our study contributes to the growing body of literature on the intersection of time perspective, emotion regulation, and mental health, emphasizing the need for culturally sensitive interventions tailored to address the complex cognitive and emotional patterns associated with dysfunctional time perspectives. Moving forward, future research should continue to explore the cultural nuances of coping strategies and their implications for mental health outcomes, with the aim of informing more effective and culturally appropriate interventions for individuals experiencing symptoms of anxiety and depression.

## Data Availability

The raw data supporting the conclusions of this article will be made available by the authors, upon reasonable request.
